# Early marker of ocular neurodegeneration in children and adolescents with type 1 diabetes: the contributing role of polymorphisms in *mir146a* and *mir128a* genes

**DOI:** 10.1007/s00592-022-01919-7

**Published:** 2022-08-25

**Authors:** Claudia Piona, Silvia Costantini, Chiara Zusi, Tiziano Cozzini, Emilio Pedrotti, Marco Marigliano, Elena Fornari, Alice Maguolo, Anita Morandi, Claudio Maffeis

**Affiliations:** 1 Section of Pediatric Diabetes and Metabolism, Department of Surgery, Dentistry, Pediatrics, and Gynecology, University of Verona, Verona, P.le Stefani 1, 37126 Verona, Italy; 2 Department of Neuroscience, Biomedicine and Movement Sciences, Eye Clinic, University of Verona, P.le L. A. Scuro 10, 37134 Verona, Italy

**Keywords:** Type 1 diabetes, Diabetic neuropathy, Ocular neurodegeneration, Genetic susceptibility,, MicroRNAs, MIR146A gene polymorphism, MIR128A gene polymorphism

## Abstract

**Background:**

Early ocular neurodegenerative signs of diabetic neuropathy (DN) can be found in children and adolescents with type 1 diabetes (T1D). No data are available on the potential role of polymorphisms in miRNAs genes in predisposing T1D subjects to these signs.

**Aims:**

To determine whether *MIR146A* rs2910164 and *MIR128A* rs11888095 polymorphisms are associated with early retinal and corneal neurodegenerative changes in pediatric patients with T1D.

**Methods:**

A total of 140 T1D children/adolescents underwent spectral domain-optical coherence tomography (SD-OCT) and in vivo confocal microscopy (IVCM) with measurement of retinal and corneal nerve fiber parameters. Risk factors for diabetes complications (diabetes duration, blood pressure, HbA1c) were recorded. Genotyping of rs2910164 and rs1188095 SNPs and genotype–phenotype association analysis were performed.

**Results:**

The C allele of rs2910164 in *MIR146A* was associated with higher values of IVCM parameters and minimum rim width (MRW) of the peripapillary region of optic nerve head measured in the retina, whereas the T allele of rs1188095 in *MIR128A* was associated with a significant impairment of them. Multiple regression analysis showed that *MIR146A* and *MIR128A* polymorphisms were significantly associated with corneal nerve fiber length (beta = 0.225 and − 0.204, respectively) and other IVCM parameters, independently from age, diabetes duration, HbA1c and systolic blood pressure percentile. Similar results were found for MRW (beta = 0.213 and − 0.286, respectively).

**Conclusions:**

These results provide new insight into the genetic predisposition to DN showing that two polymorphisms in *MIR146A* and *MIR128A* genes could significantly contribute to the development of early ocular preclinical signs of DN.

**Supplementary Information:**

The online version contains supplementary material available at 10.1007/s00592-022-01919-7.

## Introduction

Diabetic neuropathy (DN) is one of major microvascular complications of type 1 diabetes (T1D). This disease is characterized by an insidious onset. Overt symptoms usually manifest in adults when damage to nerve fibers is advanced [1]. Clinical and neurophysiological tests recommended by current guidelines are able to mainly detect gross alterations of nerve fibers [2]. For this reason, their diagnostic sensibility and utility are currently considered limited in children and youths with T1D [3]. Moreover, several studies demonstrated that early neurodegenerative alterations could occur in childhood and adolescence in the first years after the onset of T1D and involve firstly small nerve fibers [2].

In recent years, two new ophthalmologic imaging techniques, Spectral domain-optical coherence tomography (SD-OCT) and In vivo confocal microscopy (IVCM), emerged for their ability to identify early neurodegenerative changes in retina and cornea of subjects with diabetes [4]. Concerning the evaluation of these early ocular changes during the pediatric age, we demonstrated that a significant impairment of the minimum neuroretinal rim width (MRW) of the optic nerve head (ONH) measured with SD-OCT and of the corneal subbasal nerve plexus (SBP) measured with IVCM is present in T1D children and adolescents compared with age and gender-matched controls [5, 6]. Our results also showed that long-term glycometabolic control and blood pressure partially predicted the presence of early damage signs, in agreement with recent findings that recognized glycemic control and hypertension as major risk factors for DN development and progression [2].

However, the pathogenesis of DN is multifactorial and in recent years mounting evidence demonstrated that various genetic and epigenetic factors contribute to its development. In particular, a potential role in the pathogenesis of DN may be played by microRNAs (miRNAs), a family of small non-coding RNAs with enormous regulatory role in controlling posttranscriptional expression of their target genes [7].

**Fig. 1:**
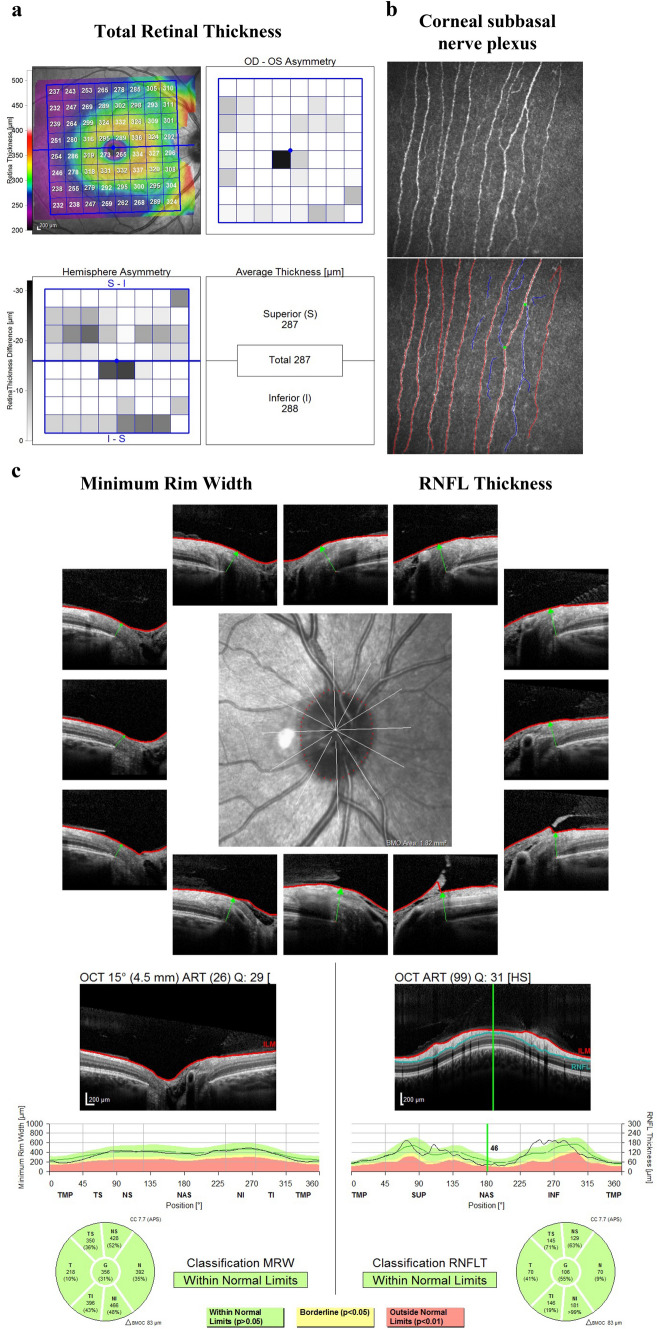
Representative images from one study participants: total retinal thickness (TRT) of the macula (panel **A**); image of the corneal subbasal nerve plexus (red, fiber; blue, branch; green, branch point) (panel **B**); Minimum Rim Width (MRW) and Retinal Nerve Fiber Layer (RNFL) of the peripapillary region of Optic Nerve Head (ONH) (panel **C**)

Their pathological down- or up-regulation is associated with chronic disease states, including diabetes and range of diabetes-associated complications that manifest with microvasculature dysfunction [8]. In particular, alterations in the expression profiles of miRNA have been found in subjects with type 2 diabetes, whereas a few studies were carried out in subjects with T1D [9]. Moreover, miRNAs were primarily evaluated for their possible key role in the pathogenesis of T1D, although a paucity of data have been published regarding their possible contribution in the development of long-term complications of T1D. Furthermore, some recent data outlined that the presence of genetic variations, such as single nucleotide polymorphisms (SNPs), in genes coding miRNA could affect their maturation and function altering expression of the gene, inducing aberrant maturation, or modifying target-binding affinity and specificity [10]. Thus, the assessment of polymorphisms in miRNA genes, in addition to the miRNA expression profiles investigation, could revealed interesting and novel insights regarding genetic susceptibility to several pathologic processes [11]. A recent study performed in adult with type 2 diabetes reported an association of two polymorphisms in *MIR146A* (rs2910164) and in *MIR128A* genes (rs11888095) with DN susceptibility [12] (Fig. 1).

To the best of our knowledge, no data are available on the potential role of polymorphisms in miRNAs genes predisposing T1D subjects to the development of early neurodegenerative signs of DN. In particular, pediatric age is ideal for conducting researches on this specific topic. In fact, at this age, it is possible to evaluate the contributing role of genetic and T1D-related factors in determining early appearance of ocular neurodegenerative signs before other acquired factors, such as environmental ones, could play their role in DN pathogenesis.

This study analyzed *MIR146A* rs2910164 and *MIR128A* rs11888095 polymorphisms in a cohort of children and adolescents with T1D, to test the hypothesis that they are associated with early retinal and corneal neurodegenerative changes.

## Methods

### Study population

This cross-sectional study was conducted at the Regional Center for Pediatric Diabetes of the University Hospital of Verona (Italy) in collaboration with the Eye Clinic, Department of Neurosciences, Biomedicine and Movement Sciences of the University of Verona. The study protocol was approved by the Institutional Ethics Committee of Verona (Italy).

One hundred and fifty children and adolescents with T1D were consecutively enrolled. Inclusion criteria were age between 10 and 22 years (both inclusive) and diagnosis of T1D for at least 2 years prior to study enrollment. Exclusion criteria were: diagnosis of diabetic retinopathy and/or neuropathy according to the current ISPAD guidelines [2], diagnosis of glaucoma, corneal and lens opacities, major refractive errors (> + 5 and < − 8 diopters sphere), history of corneal abnormality, trauma or surgery, contact lens wear or other ophthalmological disorders, and significant systemic chronic diseases other than T1D.

### Clinical and biochemical data collection

At the time of the study enrollment, all the study participants underwent a physical examination with the collection of anthropometric (body height and body weight) and blood pressure (BP) measurements. BMI was standardized calculating age and gender-specific BMI percentiles according to the WHO child growth standards [13]. Blood pressure was measured on the left arm in sitting position for three times using a digital sphygmomanometer with a cuff appropriate for children’s age and arm circumference. The average of the three measurements was recorded for the analysis and the percentile of systolic and diastolic BP (SBP and DBP) values were calculated according to gender, age and body height normative values [14].

The following demographic and clinical data were also recorded: age of onset and duration of T1D, type of insulin therapy, daily insulin dosages, type of glucose monitoring device used and HbA1c value measured with DCA Vantage® Analyzer at the enrollment visit.

### Genetic analysis

Genomic DNA was extracted from circulating leucocytes of EDTA-anticoagulated blood, using a standard salting-out procedure. Genotyping of rs2910164 and rs1188095 SNPs was performed using real-time PCR with TaqMan allelic discrimination assay (Applied Biosystems, Foster city, California, USA). Genotype distribution was in Hardy–Weinberg equilibrium for each genotyped polymorphism. Genotyping call rate was above 99% for each plate.

### Ophthalmologic, SD-OCT and CCM examination

Study participants underwent a complete ophthalmologic examination with determination of visual acuity, intraocular pressure and mydriatic fundoscopy. In vivo confocal microscopy (IVCM) (Heidelberg Retinal Tomography III with Rostock Cornea Module, Heidelberg Engineering, GE) and SD-OCT (Spectralis HRA+OCT, software version 5.4.7.0; Heidelberg Engineering. Inc.) were performed in both eyes and for each parameter the mean of right and left eyes measurements was calculated. All the examinations were performed by the same expert operator (TC) as previously described [5, 6].

In particular, in vivo confocal microscopy (IVCM) examination was performed after the administration of a drop of topical lidocaine hydrochloride 40 mg/ml (A23lfa Intes Pharmaceuticals, Casoria, Italy) and a drop of ophthalmic tear gel (Tear Gel carbomer 0.3%, Thea Pharmaceuticals, Clermont-Ferrand, France) in each eye as coupling medium between the microscope objective lens and the corneal surface. The total duration of the IVCM examination was about 2 minutes per eye. Scans of the central and paracentral cornea were acquired using a sequence module for recording one image per second. The examiner manually analyzed the acquired images focusing on subbasal nerves while scanning and adjusting the axial depth dial (image resolution 384 × 384 pixels with a 400 µm2 field of view lens) in order to achieve the best possible quality of nerve images.

After the examination, the examiner selects six images per eye for each participant choosing those with higher contrast and without artefacts. The images were then processed and five corneal nerve parameters were quantified: (1) Corneal Nerve Fiber Length (CNFL), i.e., the total length of all nerve fibers and branches (mm/mm^2^) within the scanned area (2) Corneal Nerve Fiber Density (CNFD), i.e., the total number of major nerves per square millimeter of corneal tissue (n.mm^2^), (3) Corneal Nerve Branch Density (CNBD), i.e., the number of branches emanating from all major nerve trunks per square millimeter of corneal tissue (n. mm^2^), (4) corneal Nerve Fiber Total Branch Density (CTBD), i.e., the total number of branch points/mm^2^ and (5) Corneal Nerve Fiber Fractal Dimension (CNFrD), i.e., corneal nerve fiber fractal dimension, a measure of corneal nerve complexity.

SD-OCT studies ocular microstructures measuring Minimum Rim Width (MRW) and Retinal Nerve Fiber Layer (RNFL) of the peripapillary region of Optic Nerve Head (ONH), ganglion cell layer (GCL) and total retinal thickness (TRT) of the macula.

For measurement of the MRW, 24 radial B-scans of the ONH images were used to segment 48 Bruch’s membrane opening (BMO) points, as well as the internal limiting membrane (ILM); the shortest distance between these two structures was automatically calculated. The peripapillary RNFL thickness at 3.5 mm, centered on the BMO was measured. The RNFL and MRW results were displayed in a sectorial pattern: global, temporal-superior, temporal, temporal-inferior, nasal-superior, nasal, and nasal-inferior. To obtain TRT and GCL, 30° × 25° volume scans of the macula centered on the fovea were acquired; the results were displayed in a sectorial pattern divided in total, superior, and inferior. The examination was repeated if the image quality was poor.

There were no significant differences between measurements of the left and the right eye; the average of each corneal nerve and OCT parameters from both eyes was entered in the data analysis, as previously described [6].

### Statistical analysis

All continuous variables were normally distributed and are reported as means and standard deviations, unless otherwise specified. The association of MIR146A (rs2910164) and *MIR128A* (rs1188095) with the clinical outcomes was performed under an additive model and a dominant model for the minor allele (C and T, respectively).

The genotype-phenotype association analysis was performed using Kruskall–Wallis test and t-test. OCT and IVCM parameters associated with genotypes were further evaluated through analysis of co-variance using *MIR146A* or *MIR128A* genotypes codified according to a dominant model for the minor allele (*MIR146A* :CC+GC=1 and GG=0; *MIR128A:* CT+TT=1; CC=0) and traditional risk factors for diabetes complications (age, diabetes duration, gender, HbA1c and SBP percentile) as independent variables, in order to assess the contribution of these factors in explaining the inter-individual variability of ophthalmic parameters.

Significance level for all tests was set at *p *< 0.05. Data were analyzed using SPSS version 26.0 software (SPSS, Chicago, IL, USA).

## Results

### Sample characteristics

One hundred and fifty T1D patients were enrolled and underwent ophthalmic examination. All of them provided full IVCM data, whereas OCT data and blood sample for genetic analysis were not available, respectively, in three and seven patients. Therefore, the study sample was composed of 140 patients with full clinical, ophthalmic and genetic data. No significant differences in gender and pubertal status distribution, age, anthropometric parameters, blood pressure, HbA1c and ophthalmic parameters were found by comparing patients included in the final study sample with those excluded (Supplementary Table 1). Clinical characteristics of the study sample are shown in Table 1.

**Table 1:** Clinical and demographic characteristics of the study sample

	Study sample (*n* = 140)
Gender (m/f)	72 / 68
Pubertal status (pubertal/postpubertal)	30 / 110
Age (years)	17.0 (4.9)
Age at onset (years)	7.7 (3.6)
Diabetes duration (years)	9.3 (5.5)
Body height (cm)	164.7 (10.7)
Body weight (kg)	60.5 (14.7)
BMI [kg x (m^2^)]	22.0 (3.8)
BMI [kg x (m^2^)] percentile	61.1 (26.8)
SBP (mmHg)	108.6 (8.4)
SBP (percentile)	38.0 (24.6)
DBP (mmHg)	68.4 (7.6)
DBP (percentile)	56.8 (22.5)
HbA1c (%, mmol × mol^−1^)	8.09 (0.72), 64.9 (7.9)
Total insulin × kg BW × day^−1^	0.90 (0.25)
R or short-acting I × kg BW × day^−1^	0.50 (0.17)
Long-acting I × kg BW × day^−1^	0.40 (0.11)
Total cholesterol (mmol × L^−1^, mg × dL^−1^)	
	3.91 (0.71), 151.3 (27.5)
HDL cholesterol (mmol × L^−1^, mg × dL^−1^)	1.55 (0.34), 60.2 (13.2)
LDL cholesterol (mmol × L^−1^, mg × dL^−1^)	1.98 (0.60), 76.8 (23.3)
Non-HDL cholesterol (mmol × L^−1^, mg × dL^−1^)	2.20 (0.85), 85.4 (32.8)
Triglycerides (mmol × L^−1^, mg × dL^−1^)	0.75 (0.33), 67.0 (29.5)
ACR (mg × mmol^−1^)	1.82 (1.32)

Data are shown as mean and standard deviation in brackets. Abbreviations: BMI body mass index, SBP systolic blood pressure, DBP systolic blood pressure, R regular insulin, HDL high density lipoprotein, LDL low density lipoprotein, ACR albumin/creatinine ratio

### SD-OCT and IVCM Measurements

Ophthalmologic examination showed intraocular pressure within the normal range in all study participants. Mydriatic fundoscopy did not detect signs of diabetic retinopathy. Retinal and corneal ophthalmic parameters of the study sample are shown in Table 2.

**Table 2:** Corneal nerve parameters measured with CCM, macular and optic nerve head retinal parameters measured with SD-OCT in different sectorial patterns of the study sample

	Study sample (*n* = 140)
CNFD (n/mm^2^)	24.19 (5.22)
CNBD (n/mm^2^)	27.35 (10.65)
CNFL (mm/mm^2^)	14.82 (2.63)
CTBD (n/mm^2^)	41.91 (15.64)
CNFFrD	1.485 (0.021)
TRT (µm)	299.3 (12.5)
superior TRT (µm)	299.1 (12.5)
inferior TRT (µm)	299.5 (13.0)
GCL (µm)	33.7 (2.4)
superior GCL (µm)	33.7 (2.55)
inferior GCL (µm)	33.72 (2.47)
global MRW (µm)	366.30 (60.86)
temporal superior MRW (µm)	350.32 (64.22)
temporal MRW (µm)	269.24 (50.31)
temporal inferior MRW (µm)	391.67 (67.01)
nasal superior MRW (µm)	406.19 (79.76)
nasal MRW (µm)	399.68 (73.11)
nasal inferior MRW (µm)	445.20 (78.74)
global RNFL (µm)	102.73 (9.31)
temporal superior RNFL (µm)	134.82 (18.99)
temporal RNFL (µm)	72.91 (9.21)
temporal inferior RNFL (µm)	155.90 (16.58)
nasal superior RNFL (µm)	118.06 (20.91)
nasal RNFL (µm)	84.21 (11.96)
nasal inferior RNFL (µm)	120.25 (24.22)

Data are shown as mean and standard deviation in brackets. Abbreviations: CNFL Corneal Nerve Fiber Length, CNFD Corneal Nerve Fiber Density, CNBD Corneal Nerve Branch Density, CTBD corneal Nerve Fiber Total Branch Density, CNFrD Corneal Nerve Fiber Fractal Dimension, TRT total retinal thickness, GCL ganglion cell layer thickness, MRW minimum rim width, RNFL retinal nerve fiber layer

### Genetic predisposition analysis

The results of the genotype-phenotype association analysis are shown in Table 3 and Table 4.

**Table 3:** Corneal nerve parameters measured with CCM, macular and optic nerve head retinal parameters measured with SD-OCT in different sectorial patterns in study subjects according to Mir146a genotype

	MIR146a Genotypes				
	CC (n = 9)	GC (n = 58)	GG (n = 73)	P*	CC + GC *versus* GG P**
CNFD (n/mm^2^)	23.74 (3.49)	25.47 (4.77)	23.35 (4.46)	**0.07**	**0.028**
CNBD (n/mm^2^)	29.38 (12.38)	28.37 (9.35)	24.98 (9.53)	0.12	**0.048**
CNFL (mm/mm^2^)	15.07 (1.89)	15.43 (2.45)	14.26 (2.29)	0.05	**0.011**
CTBD (n/mm^2^)	46.55 (21.19)	43.40 (14.18)	39.33 (14.57)	0.30	0.104
CNFFrD	1.49 (0.02)	1.49 (0.01)	1.48 (0.02)	**0.03**	**0.002**
TRT (µm)	302.94 (10.36)	299.98 (13.85)	298.25 (10.80)	0.48	0.31
superior TRT (µm)	304.17 (9.41)	299.58 (13.70)	299.22 (12.66)	0.42	0.65
inferior TRT (µm)	301.50 (11.62)	300.49 (14.49)	298.04 (11.10)	0.37	0.24
GCL (µm)	34.75 (2.30)	34.05 (1.95)	33.23 (2.79)	0.05	**0.031**
superior GCL (µm)	34.80 (2.56)	34.09 (2.04)	33.21 (2.91)	0.05	**0.027**
inferior GCL (µm)	34.95 (2.42)	34.05 (2.05)	33.33 (2.78)	0.06	**0.048**
global MRW (µm)	381.25 (75.07)	386.66 (58.26)	356.72 (56.46)	**0.02**	**0.005**
temporal MRW (µm)	280.10 (71.28)	282.71 (55.64)	261.48 (47.09)	0.15	**0.024**
temporal superior MRW (µm)	390.85 (90.53)	359.51 (64.13)	340.49 (59.05)	**0.04**	**0.032**
temporal inferior MRW (µm)	389.70 (83.48)	414.23 (66.52)	383.53 (63.98)	**0.038**	**0.022**
nasal MRW (µm)	413.75 (80.84)	426.62 (66.10)	389.93 (67.34)	**0.015**	**0.004**
nasal superior MRW (µm)	449.85 (80.78)	420.93 (77.46)	395.70 (77.46)	0.06	**0.029**
nasal inferior MRW (µm)	433.60 (85.48)	477.26 (76.94)	431.24 (75.73)	**0.01**	**0.004**
global RNFL (µm)	107.20 (11.02)	103.80 (7.20)	101.56 (10.62)	0.17	0.092
temporal RNFL (µm)	71.95 (13.29)	73.19 (8.16)	73.22 (9.80)	0.96	0.890
temporal superior RNFL (µm)	143.15 (21.32)	135.03 (17.63)	131.04 (20.31)	0.14	0.20
temporal inferior RNFL (µm)	154.25 (26.18)	157.27 (14.09)	153.35 (16.62)	0.31	0.22
nasal RNFL (µm)	89.50 (17.12)	85.47 (11.84)	83.43 (11.85)	0.60	0.21
nasal superior RNFL (µm)	129.50 (23.14)	119.59 (19.61)	116.49 (21.28)	0.13	0.19
nasal inferior RNFL (µm)	129.65 (28.82)	122.34 (19.97)	118.65 (27.08)	0.50	0.25

*CNFL* Corneal Nerve Fiber Length, *CNFD* Corneal Nerve Fiber Density, *CNBD* Corneal Nerve Branch Density, *CTBD* corneal Nerve Fiber Total Branch Density, *CNFrD* Corneal Nerve Fiber Fractal Dimension, *TRT* total retinal thickness, *GCL* ganglion cell layer thickness, *MRW* minimum rim width, *RNFL* retinal nerve fiber layer

Genotype–phenotype association analysis was performed using Kruskall–Wallis test* and t-test**

Bold values indicate *p* values < 0.05

**Table 4:** Corneal nerve parameters measured with CCM, macular and optic nerve head retinal parameters measured with SD-OCT in different sectorial patterns in study subjects according to MIR128a genotype

	MIR128a Genotypes				
	CC (n = 93)	CT (n = 43)	TT (n = 4)	P*	CC *versus* CT + TT P**
CNFD (n/mm^2^)	25.32 (4.40)	23.68 (5.15)	20.31 (4.98)	**0.045**	**0.028**
CNBD (n/mm^2^)	29.30 (10.80)	25.58 (8.93)	21.58 (8.05)	0.135	**0.025**
CNFL (mm/mm^2^)	15.44 (2.45)	14.19 (2.48)	13.29 (2.74)	**0.033**	**0.004**
CTBD (n/mm^2^)	44.72 (15.85)	38.68 (14.29)	39.45 (11.51)	0.115	**0.039**
CNFrD	1.49 (0.02)	1.48 (0.03)	1.47 (0.02)	**0.026**	**0.022**
TRT (µm)	298.82 (12.79)	299.50 (11.77)	302.83 (7.51)	0.79	0.69
superior TRT (µm)	299.44 (13.72)	299.59 (12.28)	304.33 (6.81)	0.67	0.84
inferior TRT (µm)	298.76 (13.46)	299.29 (11.73)	301.67 (8.81)	0.92	0.77
GCL (µm)	33.51 (2.52)	33.81 (2.52)	34.00 (1.08)	0.71	0.51
superior GCL (µm)	33.48 (2.57)	33.85 (2.76)	34.13 (1.44)	0.68	0.42
inferior GCL (µm)	33.60 (2.58)	33.84 (2.47)	34.38 (1.11)	0.66	0.54
global MRW (µm)	373.95 (56.55)	343.83 (49.24)	355.88 (36.54)	**0.02**	**0.006**
temporal MRW (µm)	271.77 (50.29)	256.81 (41.15)	243.63 (31.32)	0.23	**0.045**
temporal superior MRW (µm)	355.01 (62.97)	329.73 (46.87)	357.88 (61.65)	0.17	**0.032**
temporal inferior MRW (µm)	399.99 (65.50)	375.27 (54.60)	361.00 (44.59)	0.10	**0.020**
nasal MRW (µm)	410.72 (65.68)	370.03 (62.11)	397.25 (40.34)	**0.02**	**0.003**
nasal superior MRW (µm)	414.22 (78.17)	378.07 (62.43)	429.75 (99.80)	0.06	**0.035**
nasal inferior MRW (µm)	456.77 (78.00)	416.46 (72.56)	414.63 (68.85)	**0.03**	**0.007**
global RNFL (µm)	102.94 (9.80)	102.47 (8.21)	102.38 (12.17)	0.97	0.780
temporal RNFL (µm)	72.45 (9.39)	74.33 (8.85)	68.50 (6.28)	0.39	0.461
temporal superior RNFL (µm)	135.48 (18.66)	135.03 (18.90)	129.00 (20.40)	0.66	0.768
temporal inferior RNFL (µm)	154.33 (16.88)	158.23 (15.92)	141.38 (21.46)	0.13	0.510
nasal RNFL (µm)	84.99 (12.74)	84.67 (10.61)	83.63 (8.27)	0.98	0.850
nasal superior RNFL (µm)	118.54 (21.17)	113.36 (18.20)	126.00 (34.04)	0.52	0.33
nasal inferior RNFL (µm)	120.55 (23.81)	117.60 (22.66)	141.75 (29.61)	0.39	0.92

*CNFL* Corneal Nerve Fiber Length, *CNFD* Corneal Nerve Fiber Density, *CNBD* Corneal Nerve Branch Density, *CTBD* corneal Nerve Fiber Total Branch Density, *CNFrD* Corneal Nerve Fiber Fractal Dimension, *TRT* total retinal thickness, *GCL* ganglion cell layer thickness, *MRW* minimum rim width, *RNFL* retinal nerve fiber layer

Genotype–phenotype association analysis was performed using Kruskall–Wallis test* and t-test**

Bold values indicate *p* values < 0.05

The rs1188095 SNP (*MIR146A)* showed a significant association with CNFL, CNFrD, global, temporal superior, temporal inferior, nasal and nasal inferior MRW. Patients homozygotes for the G allele had significantly lower CCM parameters, with the exception of CTBD, and also in MRW and GCL parameters measured in all ONH and macular sectors compared to carriers of the C allele (all *p *< 0.05) (Table 3).

CNFD, CNFL, CNFrD and MRW measured in most of the ONH sectors significantly varied across the three rs2910164 *MIR128A* genotypes, whereas no significant associations were found for the other OCT parameters. In particular, patients with the *MIR128A* rs2910164 CC genotype had significantly higher values of CNFD, CNFL, CNFrD, global, temporal superior, nasal, nasal superior and nasal inferior MRW, compared to the TC and TT carriers (all p < 0.05), which, in contrast, did not differ from each other for any parameter (Table 4).

### 3.4. Multiple regression analysis

The results of the multiple regression analysis run using the main risk factors for diabetes complications measured at the time of study enrollment as dependent variables are shown in Table 5 and Table 6.

**Table 5:** Multiple regression analysis of MIR146a genotype, clinical and biochemical parameters for the risk factors for diabetes complications measured at IVCM and OCT evaluation

Dependent variable	Variables in the model	*B*	95% CI	*P*
CNFL (model *R*^2^ = 0.118, *p* = 0.040)	Age	0.105	− 0.088–0.299	0.283
	Diabetes duration	− 0.89	− 0.219–0.041	0.176
	HbA1c	− 0.534	− 1.254–0.185	0.144
	SBP percentile	− 0.002	− 0.021–0.016	0.790
	MIR146a genotypes*	1.014	0.066–1.963	*0.036*
CNFD (model *R*^2^ = 0.118, *p* = 0.041)	Age	0.163	− 0.240–0.565	0.425
	Diabetes duration	− 0.235	− 0.504–0.035	0.087
	HbA1c	− 1.090	− 2.585–0.405	0.151
	SBP percentile	0.013	− 0.025–0.051	0.486
	MIR146a genotypes*	2.029	0.057–4.00	*0.044*
CNBD (model *R*^2^ = 0.183, *p* = 0.002)	Age	0.855	0.33–1.677	0.042
	Diabetes duration	− 0.724	− 1.274– − 0.174	0.011
	HbA1c	− 2.823	− 5.873–0.227	0.069
	SBP percentile	− 0.050	− 0.127–0.027	*0.201*
	MIR146a genotypes*	3.795	− 0.228–7.818	0.064
CTBD (model R^2^ = 0.146, *p* = 0.012) CNFFrD (model *R*^2^ = 0.165, *p* = 0.005)	Age	1.287	0.100–2.574	0.040
	Diabetes duration	− 0.834	− 1.695–0.027	0.058
	HbA1c	− 3.622	− 8.396–1.151	0.135
	SBP percentile	− 0.101	− 0.222–0.020	0.101
	MIR146a genotypes*	− 4.528	− 1.768–10.823	0.157
	Age	0.001	0.00–0.003	0.139
	Diabetes duration	− 0.001	− 0.002–0.000	0.143
	HbA1c	− 0.005	− 0.12–0.001	0.111
	SBP percentile	0.0	0.0	0.933
	MIR146a genotypes*	0.12	0.003–0.021	*0.007*
global MRW (model *R*^2^ = 0.128, *p* = 0.013)	Age	1.847	− 2.918–6.612	0.444
	Diabetes duration	− 2.099	− 5.371–1.174	0.206
	HbA1c	− 17.311	− 34.177– − 0.446	0.044
	SBP percentile	0.266	− 0.176–0.707	0.236
	MIR146a genotypes*	26.002	2.104– − 49.899	0.033

*CNFL* Corneal Nerve Fiber Length, *CNFD* Corneal Nerve Fiber Density, *CNBD* Corneal Nerve Branch Density, *CTBD* corneal Nerve Fiber Total Branch Density, *CNFrD* Corneal Nerve Fiber Fractal Dimension, *MRW* minimum rim width

^*^MIR146A genotypes were codified according to a dominant model for the minor allele C: CC + GC = 1 and GG = 0

**Table 6:** Multiple regression analysis of MIR128a genotype, clinical and biochemical parameters for the risk factors for diabetes complications measured at IVCM and OCT evaluation

Dependent variable	Variables in the model	*B*	95% CI	*P*
CNFL (model *R*^2^ = 0.116, *p* = 0.033)	Age	0.059	− 0.153–0.271	0.583
	Diabetes duration	0.035	− 0.111–0.271	0.640
	HbA1c	− 0.290	− 0.993 − 0.413	0.415
	SBP percentile	− 0.016	− 0.035–0.002	0.087
	MIR128a genotypes*	− 1.048	− 2.087– − 0.010	*0.038*
CNFD (model *R*^2^ = 0.090, *p* = 0.042)	Age	0.084	− 0.356–0.524	0.706
	Diabetes duration	0.059	− 0.244–0.361	0.701
	HbA1c	− 0.473	− 1.931–0.984	0.521
	SBP percentile	− 0.010	− 0.049–0.029	0.597
	MIR128a genotypes*	− 1.981	− 3.977– − 0.015	*0.042*
CNBD (model *R*^2^ = 0.148, *p* = 0.008)	Age	0.766	− 0.131–1.664	0.094
	Diabetes duration	− 0.585	− 1.203–0.033	0.063
	HbA1c	− 2.578	− 5.553–0.397	0.089
	SBP percentile	− 0.085	− 0.164– − 0.05	*0.037*
	MIR128a genotypes*	− 2.691	− 7.085–1.703	0.227
CTBD (model R^2^ = 0.161, *p* = 0.003)	Age	1.139	− 0.181–2.459	0.090
	Diabetes duration	− 0.588	− 1.496–0.320	0.202
	HbA1c	− 2.659	− 7.032–1.713	0.230
	SBP percentile	− 0.156	− 0.274– − 0.039	**0.010**
	MIR128a genotypes*	− 6.856	− 13.042– − 0.671	**0.030**
CNFFrD (model *R*^2^ = 0.160, *p* = 0.004)	Age	0.001	− 0.001–0.003	0.231
	Diabetes duration	0.0	− 0.001–0.001	0.965
	HbA1c	− 0.004	− 0.10–0.002	0.216
	SBP percentile	0.0	0.0	0.070
	MIR128a genotypes*	− 0.10	− 0.019–0.001	**0.025**
global MRW (model *R*^2^ = 0.134, *p* = 0.014)	Age	1.190	− 3.157–5.537	0.588
	Diabetes duration	− 1.810	− 4.988–1.368	0.261
	HbA1c	− 14.828	− 30.463–0.806	0.063
	SBP percentile	0.278	− 0.152–0.708	0.202
	MIR128a genotypes*	− 35.117	− 58.885– − 11.350	**0.004**

*CNFL* Corneal Nerve Fiber Length, *CNFD* Corneal Nerve Fiber Density, *CNBD* Corneal Nerve Branch Density, *CTBD* corneal Nerve Fiber Total Branch Density, *CNFrD* Corneal Nerve Fiber Fractal Dimension, *MRW* minimum rim width

^*^MIR128A genotypes were codified according to a dominant model for the minor allele T: CT + TT = 1; CC=0

Bold values indicate *p* values < 0.05

*MIR146A* polymorphism was significantly associated with variability of CNFL (B coefficient = 1.014, *p *= 0.036, beta coefficient = 0.225), CNFD (B coefficient=2.029, *p *= 0.044, beta coefficient= 0.216 and CNFrD (B coefficient =0.12, *p *= 0.007, beta coefficient = 0.285).

*MIR146A* polymorphism was not significantly associated with CTBD and CNBD values.

Similar results were found for multiple regression analysis run using *MIR128A* (rs2910164) polymorphism (Table 6). In particular, *MIR128A* (rs2910164) polymorphism was significantly associated with CNFL (B coefficient  = − 1.048, *p *= 0.038, beta coefficient= − 0.204), CNFD (B coefficient  = − 1.981, *p *= 0.042, beta coefficient= − 0.192) and CNFFrD (B coefficient = − 0.10, *p *= 0.025, beta coefficient = − 0.226), independently from age, diabetes duration, HbA1c and SBP. *MIR128A* polymorphism (B coefficient = − 6.856, *p *= 0.030, beta coefficient = − 0.209) and SBP percentile (B coefficient = − 0.156, *p *= 0.046, beta coefficient = − 0.250) were significantly associated with CTBD (overall *R*^2 ^= 0.010), whereas *MIR128A* polymorphism was not significantly associated with CNBD (Table 5).

Regarding OCT parameters, both *MIR146A* polymorphism (B coefficient = 26.002, *p *= 0.033, beta coefficient = 0.213) and HbA1c (B coefficient = − 17.31, *p *= 0.044, beta coefficient = − 0.199), were associated to global MRW (overall *R*^2 ^= 0.128), independently from the other variables. Moreover, *MIR128A* genotype was significantly associated with global MRW (B coefficient = − 35.117, *p *= 0.004, beta coefficient = − 0.286).

## Discussion

The main result of this study was the evidence that two polymorphisms within *MIR146A* and *MIR128A* were significantly associated with early retinal and corneal neurodegenerative changes detected with SD-OCT and IVCM in children and adolescents with T1D.

To the best of our knowledge, this is the first study investigating the association between genetic predisposition and early alterations of small nerve fibers of the retina and the cornea detectable using two new ophthalmologic imaging techniques.

In two previous studies, we applied these techniques showing that corneal and retinal alterations are present in children and adolescents with T1D compared to healthy peers [5, 6]. Moreover, we also evaluated the role of classical risk factors for diabetes complications demonstrating that the presence of early ocular neurodegenerative signs in our cohort was partially predicted by worse long-term glycometabolic control and high blood pressure.

In this study we hypothesized that polymorphisms in miRNAs might also significantly contribute to the susceptibility to early ocular preclinical signs of DN.

Our results demonstrate that polymorphisms in *MIR146A* and *MIR128A* genes, in addition to glycemic control and blood pressure, significantly contribute to explain the interindividual variability of CCM and MRW parameters.

In particular, the C allele of rs2910164 SNP in *MIR146A* was associated with higher values of CCM and MRW parameters suggesting that this variant could have a protective effect against the development of early ocular neurodegenerative changes. In recent years, two studies evaluated the role of this allele in relation to the risk of diabetes microvascular complications reporting conflicting results. Kaidonis *et al.* demonstrated that C allele is associated with an increased risk of diabetic nephropathy in adults with T1D, whereas not significant association were found with diabetic retinopathy or diabetic macular edema [15]. Ciccaci *et al.* showed that C allele significantly contributed to both diabetic polyneuropathy and cardiovascular autonomic neuropathy [12]. These findings are in agreement with the results of our study. From the pathogenic point of view, it has been demonstrated that the presence of the C allele leads to a significant increase of the expression of miR146a and, consequently, a deregulation of its action on several target genes [16]. In particular, higher miR146a expression could reduce NF-kb activity and, thus, the expression of several inflammatory cytokines involved in NF-kb-mediated inflammation [17, 18]. Several recent studies outlined that inflammation is one of the most relevant pathogenetic pathway for the development and progression of DN [19]. In particular, a significant correlation have been found between higher level of proinflammatory cytokines (i.e. IL-6, IL-1, tumor necrosis factor (TNF)-a and transforming growth factor-b), whose production is induced by NF-κB, and the progression of nerve degeneration [20].

Our results also showed that T allele of rs1188095 SNP in *MIR128A* was associated with a significant impairment of MRW and corneal nerve fiber parameters. Limited data are currently available regarding the possible consequences of dysregulation of mir128A expression, however among the miR-128 targets several important proteins, such as Reelin, DCX and SNAP 25, involved in neuronal cells differentiations, migration, dendritic growth and branching, synaptogenesis and synaptic plasticity, have been identified [21]. Further studies are needed to better understand the pathogenetic role of *MIR128A* in DN and to confirm the association between the rs1188095 SNP variant and the development of early ocular neurodegenerative signs in people with diabetes.

This study has some limitations: (1) the sample size is relatively modest, although post hoc power analysis showed that the sample size of subjects grouped according to *MIR146A* and *MIR128a* genotypes following dominant model for the minor allele (n. 73 *vs.* 67 and 93 *vs.* 47, respectively) allows to detect a minimal difference in ONH and CCM parameters between genotypes groups equal to 0.42 and 0.44 standard deviation, respectively, with a statistical power of at least 80% and alpha error probability of 5%; (2) the study was conducted in subjects of European ancestry, not allowing the evaluation of risk alleles in subjects with other ancestry.

The strengths of this study are: (1) the use of both SD-OCT and CCM to examine both eyes and accurately measure several parameters acknowledge as biomarkers of early ocular neurodegeneration and, thus, early preclinical signs of DN; (2) the contemporaneous analysis of genetic predisposition driven by miRNA genes and clinical risk factors for diabetes complications.

In conclusion, this study demonstrated that two polymorphisms in *MIR146A* and *MIR128A* genes are associated with early ocular neurodegenerative changes in children and youths with T1D. These results provide new insight into the genetic predisposition to DN showing that these polymorphisms, together with long-term glycometabolic control and blood pressure, could significantly contribute to the development of early ocular preclinical signs of DN. The identification of subjects with T1D genetically predisposed to the development of ocular neurodegenerative changes is particularly valuable both clinical practice and research setting. Indeed, the prompt recognition of early ocular preclinical signs of DN in high-risk subjects could allow an early intervention on known risk factors. Moreover, despite recent advances in the treatment of DN symptoms, currently there are no treatment options able to influence its natural history targeting specific pathogenetic mechanisms. Thus, the recognition of subjects with T1D at higher risk of developing early neurodegeneration changes could also favor the identification of new pathogenetic mechanisms and therapeutic targets.

Further studies in larger cohorts of people with T1D are needed to confirm these associations and to search for other genetic factors possibly contributing to the susceptibility to early ocular preclinical signs of DN.

## Supplementary Information

Below is the link to the electronic supplementary material.

Supplementary file1 (DOCX 43 kb)

## Abbreviations

DR: Diabetic retinopathy
T1D: Type 1 diabetes
SD-OCT: Spectral Domain Optical coherence tomography
IVCM: In vivo confocal microscopy
MRW: Minimum neuroretinal rim width
ONH: Optic nerve head
SBP: Subbasal nerve plexus
miRNAs: MicroRNAs
SNPs: Single nucleotide polymorphisms
BP: Blood pressure
CNFL: Corneal Nerve Fiber Length
CNFD: Corneal Nerve Fiber Density
CNBD: Corneal Nerve Branch Density
CTBD: Corneal Nerve Fiber Total Branch Density
CNFrD: Corneal Nerve Fiber Fractal Dimension
RNFL: Retinal nerve fiber layer
GCL: Ganglion cell layer
TRT: Total retinal thickness
ANOVA: Analysis of variance
