# Supplementary material for: Early marker of ocular neurodegeneration in children and adolescents with type 1 diabetes: the contributing role of polymorphisms in mir146a and mir128a genes
Source: Acta Diabetol. 2022 Aug 25;59(12):1551–61. doi: 10.1007/s00592-022-01919-7 (PMC9581843; doi:10.1007/s00592-022-01919-7)
Supplement: Supplementary file 1 — Supplementary file1 (DOCX 43 kb) [file 592_2022_1919_MOESM1_ESM.docx]

**Supplementary material**

Supplemental table 1. Clinical, demographic biochemical, CCM and SD-OCT parameters and genotype distribution subjects include in the final study sample and excluded subjects.

|  | **Study sample** **(n= 140)** | **Excluded subjects (n=10)** | ***P*** | **Total sample (n=150)** |
| --- | --- | --- | --- | --- |
| **Gender (m/f)** | 72 / 68 | 5/5 | 0.93 | 77 / 73 |
| **Pubertal status (pubertal/postpubertal)** | 30 / 110 | 4/6 | 0.17 | 34 / 116 |
| **Age (years)** | 17.0 (4.9) | 15.9 (4.1) | 0.41 | 16.6 (4.0) |
| **Age at onset (years)** | 7.7 (3.6) | 7.3 (4.1) | 0.68 | 7.5 (3.6) |
| **Diabetes duration (years)** | 9.3 (5.5) | 8.5 (4.2) | 0.59 | 8.7 (4.2) |
| **Height (cm)** | 164.7 (10.7) | 161.9 (11.7) | 0.36 | 164.7 (10.8) |
| **Weight (kg)** | 60.5 (14.7) | 59.3 (17.5) | 0.77 | 60.5 (14.5) |
| **BMI [kg x (m^2^)]** | 22.0 (3.8) | 22.1 (4.2) | 0.90 | 22.0 (3.8) |
| **BMI [kg x (m^2^)] percentile** | 61.1 (26.8) | 62.2 (29.5) | 0.67 | 61.2 (27.4) |
| **SBP (mmHg)** | 108.6 (8.4) | 109.3 (12.4) | 0.49 | 108.9 (8.7) |
| **SBP (percentile)** | 38.0 (24.6) | 42.1 (29.5) | 0.15 | 38.1 (25.2) |
| **DBP (mmHg)** | 68.4 (7.6) | 69.5 (7.8) | 0.58 | 68.8 (7.8) |
| **DBP (percentile)** | 56.8 (22.5) | 59.2 (21.8) | 0.59 | 57.0 (22.4) |
| **HbA1c (%,mmol x mol^-1^)** | 8.09 (0.72), 64.9 (7.9) | 8.17 (0.75) | 0.55 | 8.07 (0.71); 65 (5.7) |
| **Total insulin x kg BW x day^-1^** | 0.90 (0.25) | 0.89 (0.24) | 0.96 | 0.90 (0.24) |
| **R or short-acting I x kg BW x day^-1^** | 0.50 (0.17) | 0.49 (0.18) | 0.42 | 0.50 (0.17) |
| **Long-acting I x kg BW x day^-1^** | 0.40 (0.11) | 0.42 (0.13) | 0.61 | 0.40 (0.11) |
| **Total cholesterol (mmol x L^-1^, mg x dL^-1^)** | 3.91 (0.71), 151.3 (27.5) | 3.82 (0.70), 147.8 (27.3) | 0.64 | 3.9 (0.75), 151.5 (28.8) |
| **HDL cholesterol (mmol x L^-1^, mg x dL^-1^)** | 1.55 (0.34), 60.2 (13.2) | 1.44 (0.30), 55.5 (11.4) | 0.16 | 1.53 (0.34); 59.6 (13.2) |
| **LDL cholesterol (mmol x L^-1^, mg x dL^-1^)** | 1.98 (0.60), 76.8 (23.3) | 2.03 (0.65), 78.8 (23.8) | 0.76 | 2.0 (0.64); 77.5 (24.8) |
| **Non-HDL cholesterol (mmol x L^-1^, mg x dL^-1^)** | 2.20 (0.85), 85.4 (32.8) | 2.27 (0.87) , 88.1 (33.9) | 0.74 | 2.3 (0.79), 89.0 (30.9) |
| **Triglycerides (mmol x L^-1^, mg x dL^-1^)** | 0.75 (0.33), 67.0 (29.5) | 0.77 (0.23), 68.2 (20.8) | 0.58 | 0.76 (0.33); 67.6 (30.0) |
| **ACR (mg x mmol^-1^)** | 1.82 (1.32) | 1.87 (1.40) | 0.45 | 1.85 (1.62) |
| **CNFD (n/mm^2^)** | 24.19 (5.22) | 22.70 (6.83) | 0.31 | 23.77 (5.43) |
| **CNBD (n/mm^2^)** | 27.35 (10.65) | 24.03 (12.99) | 0.26 | 26.75 (12.34) |
| **CNFL (mm/mm^2^)** | 14.82 (2.63) | 13.18 (3.83) | 0.12 | 14.51 (2.87) |
| **CTBD (n/mm^2^)** | 41.91 (15.64) | 36.42 (18.81) | 0.20 | 40.63 (16.59) |
| **CNFFrD** | 1.485 (0.021) | 1.464 (0.044) | 0.074 | 1.481 (0.026) |
| **TRT (µm)** | 299.3 (12.5) | 294.6 (10.6) | 0.13 | 299.0 (12.9) |
| **superior TRT (µm)** | 299.1 (12.5) | 2932.9 (10.4) | 0.099 | 299.5 (13.6) |
| **inferior TRT (µm)** | 299.5 (13.0) | 295.2 (11.2) | 0.19 | 298.9 (13.3) |
| **GCL (µm)** | 33.7 (2.4) | 33.0 (3.5) | 0.42 | 33.51 (2.47) |
| **superior GCL (µm)** | 33.7 (2.55) | 33.0 (3.65) | 0.41 | 33.52 (2.60) |
| **inferior GCL (µm)** | 33.72 (2.47) | 33.31 (3.42) | 0.61 | 33.56 (2.48) |
| **global MRW (µm)** | 366.30 (60.86) | 360.01 (59.40) | 0.23 | 360.09 (55.93) |
| **temporal MRW (µm)** | 269.24 (50.31) | 259.72 (47.12) | 0.16 | 264.54 (47.60) |
| **temporal superior MRW (µm)** | 350.32 (64.22) | 339.11 (58.21) | 0.22 | 340.84 (55.96) |
| **temporal inferior MRW (µm)** | 391.67 (67.01) | 384.82 (60.42) | 0.067 | 385.42 (61.54) |
| **nasal MRW (µm)** | 399.68 (73.11) | 387.50 (69.11) | 0.082 | 394.54 (69.66) |
| **nasal superior MRW (µm)** | 406.19 (79.76) | 395.41 (73.92) | 0.068 | 398.43 (74.15) |
| **nasal inferior MRW (µm)** | 445.20 (78.74) | 431.33 (77.42) | 0.0.61 | 437.48 (77.82) |
| **global RNFL (µm)** | 102.73 (9.31) | 99.73 (8.89) | 0.25 | 102.07 (8.99) |
| **temporal RNFL (µm)** | 72.91 (9.21) | 72.98 (9.25) | 0.74 | 72.94 (9.13) |
| **temporal superior RNFL (µm)** | 134.82 (18.99) | 125.50 (18.67) | 0.081 | 133.40 (18.87) |
| **temporal inferior RNFL (µm)** | 155.90 (16.58) | 146.26 (18.75) | 0.46 | 154.86 (17.09) |
| **nasal RNFL (µm)** | 84.21 (11.96) | 83.43 (11.55) | 0.81 | 83.71 (11.18) |
| **nasal superior RNFL (µm)** | 118.06 (20.91) | 116.13 (19.17) | 0.73 | 116.58 (19.36) |
| **nasal inferior RNFL (µm)** | 120.25 (24.22) | 115.33 (26.44) | 0.46 | 119.23 (24.39) |

Data are expressed as mean and standard deviation in brackets.

Abbreviations: BMI body mass index, SBP systolic blood pressure, DBP systolic blood pressure, R regular insulin, HDL high density lipoprotein, LDL low density lipoprotein, ACR albumin/creatinine ratio, CNFL Corneal Nerve Fiber Length, CNFD Corneal Nerve Fiber Density, CNBD Corneal Nerve Branch Density, CTBD corneal Nerve Fiber Total Branch Density, CNFrD Corneal Nerve Fiber Fractal Dimension, TRT total retinal thickness, GCL ganglion cell layer thickness, MRW minimum rim width, RNFL retinal nerve fiber layer.
